# The Time Course of Dorsal and Rostral-Ventral Anterior Cingulate Cortex Activity in the Emotional Stroop Experiment Reveals Valence and Arousal Aberrant Modulation in Patients with Schizophrenia

**DOI:** 10.1007/s10548-018-0677-0

**Published:** 2018-10-04

**Authors:** F. S. Feroz, G. Leicht, J. Rauh, C. Mulert

**Affiliations:** 1 Psychiatry Neuroimaging Branch (PNB), Department of Psychiatry and Psychotherapy, University Medical Center Hamburg - Eppendorf, 20246 Hamburg, Germany; 2 Center for Telecommunication Research and Innovation (CeTRI), Fakulti Kejuruteraan Elektronik dan Kejuruteraan Komputer (FKEKK), Universiti Teknikal Malaysia Melaka (UTeM), Malacca, Malaysia; 3 Centre for Psychiatry and Psychotherapy, Justus Liebig University, Giessen, Germany

**Keywords:** Schizophrenia, Emotion–cognition, Dorsal anterior cingulate cortex, Rostral-ventral anterior cingulate cortex, Arousal, Valence

## Abstract

This paper aims to investigate the temporal dynamics within the dorsal anterior cingulate cortex (dACC) and the rostral-ventral (rv) ACC during the interaction of emotional valence and arousal with cognitive control in patients with Schizophrenia (SZ). Although cognitive deficits in SZ are highly relevant and emotional disturbances are common, the temporal relationship of brain regions involved in the interaction of emotional and cognitive processing in SZ is yet to be determined. To address this issue, the reaction time (RT), event-related potential (ERP) and temporal dynamics of the dACC and rvACC activity were compared between SZ subjects and healthy controls (HC), using a modified emotional Stroop experiment (with factors namely congruence, arousal and valence). EEG was recorded with 64 channels and source localisation was performed using the sLORETA software package. We observed slower initial increase and lower peaks of time course activity within the dACC and rvACC in the SZ group. In this particular group, the dACC activity during late negativity was negatively correlated with a significantly higher RT in the high arousal conflict condition. In contrast to HC subjects, at the N450 window, there was no significant valence (ERP and rvACC ROI) modulation effect in the SZ subjects. Using high density EEG and source localisation, it was possible to distinguish various disturbances within the dACC and rvACC in patients with SZ, during emotion–cognition processing.

**Electronic supplementary material:**

The online version of this article (10.1007/s10548-018-0677-0) contains supplementary material, which is available to authorized users.

## Introduction

While being consumed with unmet daily necessities, patients suffering from Schizophrenia (SZ) are usually left with little to no capacity to handle highly charged emotional-cognitive situations (Myin-Germeys et al. 2005; Watson 2015). Cognitive deficits in patients with SZ have received a lot of attention, as it should be relevant to the functional outcome (Bowie and Harvey 2006; Soria et al. 2018). However, despite recent advancements, antipsychotic medication (Carpenter and Koenig 2008; Feifel et al. 2016; Howes et al. 2017) and cognitive enhancement therapy (Fakra et al. 2015) thus far has only shown limited impact towards the functional outcome of patients with SZ. It has been suggested that pure cognitive processes, such as those evaluated and trained in numerous programs may be distant from real-world applicability (Wykes et al. 2011).

Gjerde theorised that investigating the “cold cognitive” brain regions while precluding the “hot affective” element in SZ research could be misleading (Gjerde 1983). Cognitive and affective components were previously viewed as components that would impact separate brain regions (Bush 2000; Mohanty et al. 2007; Perlman and Pelphrey 2010). However, current research indicates the existence of interrelation between cognitive and emotional processes within the brain (Etkin et al. 2011; Inzlicht et al. 2015; Pessoa 2017). Anatomical key structures in this context are the dorsal anterior cingulate cortex (dACC) and rostral-ventral (rv) ACC (Allman et al. 2006; Stevens et al. 2011; To et al. 2017). Meanwhile, the well-established separate modulatory effects of emotional valence and arousal on behavioural and physiological responses (Vrana et al. 1988; Hempel et al. 2007; Llerena et al. 2012; Padmala et al. 2018) is of particular concern, as it influences different brain regions (Anders et al. 2004; Dolcos et al. 2004; Sieger et al. 2015) at relatively separate stages (Olofsson et al. 2008; Gianotti et al. 2008; Gallant and Dyson 2016).

The neural diathesis–stress model and its extended versions (Nuechterlein and Dawson 1984; Walker and Diforio 1997; Jones and Fernyhough 2007; Walker et al. 2008; Pruessner et al. 2017) theorised that emotional stress could trigger psychosis in vulnerable individuals. Additionally, emotional arousal activates cortisol release in humans (Cahill and McGaugh 1998). High levels of cortisol is also associated with increased arousal (Dabbs and Hopper 1990; Abercrombie et al. 2005). SZ subjects are prone to experience higher levels of cortisol and dysregulation of this stress hormone (Walder et al. 2000; Yılmaz et al. 2007; Bradley and Dinan 2010; Steen et al. 2011). Increased levels of cortisol affects the hypothalamic–pituitary–adrenal (HPA) axis by reducing hippocampus volume (Herbener et al. 2007; Mondelli et al. 2010), significantly reducing amygdala (Buckley 2005) and the amygdala–hippocampal complex volume in SZ subjects (Shenton et al. 2001). Research has shown that existing dysconnectivity between ACC and hippocampus (Cui et al. 2015); and dACC and amygdala (Liu et al. 2014) leads to behavioural deficits in SZ subjects (Williams et al. 2004; Das et al. 2007).

The dACC, amygdala and hippocampus are assumed to be part of a network system within the HPA axis that also regulates emotional arousal (Fuchs et al. 1985; Heckers and Konradi 2002; Östlund et al. 2003; Goldstein et al. 2005; Kober et al. 2008; Kanbara and Fukunaga 2016). The HPA axis is found to be disturbed in SZ subjects with severe negative symptoms (Kaneko et al. 1992). The connection between emotional arousal, stress and HPA dysfunction would relate SZ subjects to arousal dysregulation within the dACC, besides being associated with cognitive impairment. Nonetheless, the time window of this crucial state within an emotion–cognition study has yet to be determined.

The rvACC is connected to the ventral striatum (Ongür and Price 2000), nucleus accumbens (Nacc) (Ongür and Price 2000), anterior insula (Yu et al. 2011) and orbitofrontal cortex (OBF) (Ongür and Price 2000; Yu et al. 2011); all well-established components of the salience network. The DA hypothesis of SZ associates striatal hyperdopaminergia with salience detection in non-salient objects (Kapur 2003; Howes and Kapur 2009). Attentional selection is determined by the salience of a stimulus and emotional valence is a determinant for salience (Niu et al. 2012).

A caveat to the current SZ literature are inconsistent findings on the relation of emotional valence with cognitive impairment and the dysfunction of the rvACC. For instance, negative valenced items are found to promote cognitive impairment in SZ subjects (Fear et al. 1996; Mohanty et al. 2005; Habel et al. 2010b). Herbener et al. (2007), on the other hand, found effects to be statistically non-significant. SZ subjects demonstrated rvACC hypoactivity during errors of commission task (Laurens et al. 2003; Polli et al. 2008), related to affective dysfunction (Bates et al. 2002; Laurens et al. 2003; Polli et al. 2008). While violent SZ male subjects experience rvACC hyperactivations when viewing negative images, non-violent male SZ and healthy controls (HC) subjects had non-significant rvACC activation difference (Dumais et al. 2016). Mohanty et al. (2005) also found statistically non-significant differences in the modulation of rvACC activity between schizotypy and HC subjects in the negative valence condition. The variation in these findings could probably be explained by factoring in the two dimensional valence and arousal elements in experimental paradigms.

Patients with SZ are associated with arousal and valence related cognitive deficiencies. For example, attentional deficits (Gjerde 1983; Nakamura et al. 2003) are associated with the state of hyperarousal in these subjects. Individuals with SZ also face attentional and memory impairments (Walsh-Messinger et al. 2014; Yang et al. 2018) when presented with positive and negative emotional stimuli in experimental settings. The time course analysis illustrates the dynamics within the dACC and rvACC which is pivotal in understanding the effect of emotional valence and arousal during cognitive control. This provides the opportunity of detecting any arousal and valence related conflict deficiencies within specified regions of SZ subjects. This study would address the identified gaps and conflicts of previous studies by investigating the impact of emotional valence and arousal on cognitive control within the dACC and rvACC in patients with SZ, which may, in turn, lead to the discovery of the brain regions involved in aberrant emotion–cognition modulations. The objective of this study, therefore, is to unravel the underlying differences of the impact of emotional valence and arousal during cognitive control on the RT, event-related potential (ERP) and the time course of the dACC and rvACC activity in SZ subjects, in comparison to HC subjects in the emotion–cognition Stroop task.

In general, emotional valence and arousal has been found to modulate various time windows during emotion–cognition interaction. In a Go/No-Go emotion–cognition ERP task, Albert et al. (2010) found that emotional valence modulated the N200 within the ACC. On the other hand, the No-Go P3 showed differential modulation of emotional arousal in HC and patients with ADHD (López-Martín et al. 2015). In an emotion–cognition oddball-like task (Delplanque et al. 2006), the P3a modulated emotional valence while the P3b modulated emotional arousal. In an emotion–cognition Flanker study, positive and negative emotions modulated the N200 window (Kanske and Kotz 2010a, 2011a).

In the emotion–cognition Stroop study with healthy subjects, it was observed that emotional valence modulated the rvACC activity at the N450 window, reflecting initial selective attention towards emotional word valence. Notwithstanding, emotional arousal modulated the dACC activity during late negativity where emotional arousal likely initiated response conflict resolution. Based on the findings of Feroz et al. (2017) above, SZ subjects would hypothetically show deficits in modulating dACC activity at the late negativity window, interfering with conflict resolution in the high arousal condition. As previous literature suggest, evidence were found to relate SZ subjects with arousal dysregulation within the dACC, which is associated with cognitive impairment.

Inconsistent findings on the relation of emotional valence with cognitive impairment and the dysfunction of the rvACC provides a basis for the next hypothesis. This study postulates deficits in the modulation of emotional valence within the rvACC at the N450 window in SZ subjects. The study has been designed to obtain new insights and perspectives into detecting valence-conflict and arousal-conflict related deficiencies within specified regions in patients with SZ.

## Materials and Methods

### Participants

Twenty patients with SZ and 20 HC were included in the study. Patients that met the DSM-IV criteria for SZ were recruited through the Psychosis Center of the Department of Psychiatry, University Medical Center Hamburg-Eppendorf. On the other hand, HC subjects were recruited via the internet as well as word-of-mouth from Hamburg and its surrounding area. A written informed consent was obtained from all the participants following having explained the aim of the study and the nature of the procedures in full.

Exclusion criteria for all participants included any current substance abuse or dependence, presence of any major somatic or neurological disorders, colour blindness, history of reading disorder and history of intellectual disability. For HC subjects, an additional exclusion criteria of any previous psychiatric disorder or treatment was included. Handedness was assessed with the German version of the Edinburgh Handedness Manual (Oldfield 1971). All the participants had normal to corrected-to-normal vision and were native speakers of the German language. The presence of the inclusion and exclusion criteria among patients were assessed by a clinical psychiatrist or psychiatric trainee.

The Mehrfachwahl-Wortschatz-Intelligenztest (MWT-B) (Lehrl 2005), a German multiple choice vocabulary intelligence test is a valid assessment intended to measure a test-taker’s verbal crystallized intelligence. The test contains 37 items. SZ subjects were asked (without time restrictions) to identify one correct word in a list of five words (containing one real word and four pseudowords). Every correct answer was coded with 1, wrong or missing answers were coded with 0. The maximum score of the MWT-B test is 37.

The severity of clinical symptomatology was assessed with the Positive and Negative Syndrome Scale (PANSS; Kay et al. 1987). The scoring for positive, negative, disorganization, excitement and distress symptoms were created according to a five factor model of the PANSS (van der Gaag et al. 2006) and were calculated as follows: positive factor = P1 + P3 + G9 + P6 + P5 + G1 + G12 + G16 − N5; negative factor = N6 + N1 + N2 + N4 + G7 + N3 + G16 + G8 + G13 − P2; disorganization factor = N7 + G11 + G10 + P2 + N5 + G5 + G12 + G13 + G15 + G9; excitement factor = G14 + P4 + P7 + G8 + P5 + N3 + G4 + G16 and emotional distress factor = G2 + G6 + G3 + G4 + P6 + G1 + G15 + G16. Based on the reported trajectories of antipsychotic treatment response (Case et al. 2011; Stauffer et al. 2011), clinical severity ratings were used for analyses only if they were separated from EEG analyses by no more than a week. In sum, appropriate clinical ratings were available for 18 patients.

At the time of the EEG recording, nine patients were under treatment with the atypical antipsychotics while two others with typical antipsychotics. Moreover, four patients under treatment were on antidepressants and one on mood stabilizer. Additionally, ten patients were not under any psychotropic medication. None of the subjects received benzodiazepines or anticholinergic agents. The groups were then matched with respect to age, sex, and educational level. It was found that there were no significant differences (t(35) = − 1.78, *p* = .16) in the cigarette consumption (number of cigarettes per day) between the HC group and the SZ group. The demographic characteristics of the groups and clinical characteristics of the SZ participants are presented in Table 1.

**Table 1:** Participant demographic and clinical characteristics

	Healthy controls		Schizophrenia patients		*T*/*χ*^2^	*p*
	*N* or mean	SD	*N* or mean	SD		
Gender (m/f)	14/6		14/6		0.00	1.00
Age	32.00	9.38	32.65	9.78	0.21	0.83
Level of education	2.65	0.49	2.35	0.75	0.69	0.71
Handedness (R/L)	19/1		18/2		1.05	0.30
Medication dose chlorpromazine equivalent (mg/day)			232.5	325.36		–
Duration of illness (years)			9.4	6.57		–
MWT-B			26.57	4.94		–
Cigarette consumption (number/day)	2.63	6.15	7.00	8.63	− 1.78	0.16
Five-factor PANSS scores						
Positive symptoms			11.39	6.17		–
Negative symptoms			12.28	6.52		–
Disorganization			12.33	4.00		–
Excitement			9.56	2.23		–
Emotional distress			11.83	4.02		–
Total PANSS			42.44	11.04		–

### Paradigm and Task

The stimuli of this study were adapted from Chajut et al. (2010), while tasks and procedures on the other hand were adapted from Feroz et al. (2017). The stimuli consisted of an emotional word and a colour word, horizontally aligned and appeared to the left and right of a fixation point. The ink colour of the emotional word either matched (congruent) to the colour word or was mismatched (incongruent) to the colour word. Selected emotional words (Võ et al. 2009; Kanske and Kotz 2010b, 2011b) were categorized into three different valence classes (neutral, positive and negative) and two different arousal classes (high and low).

**Fig. 1:**
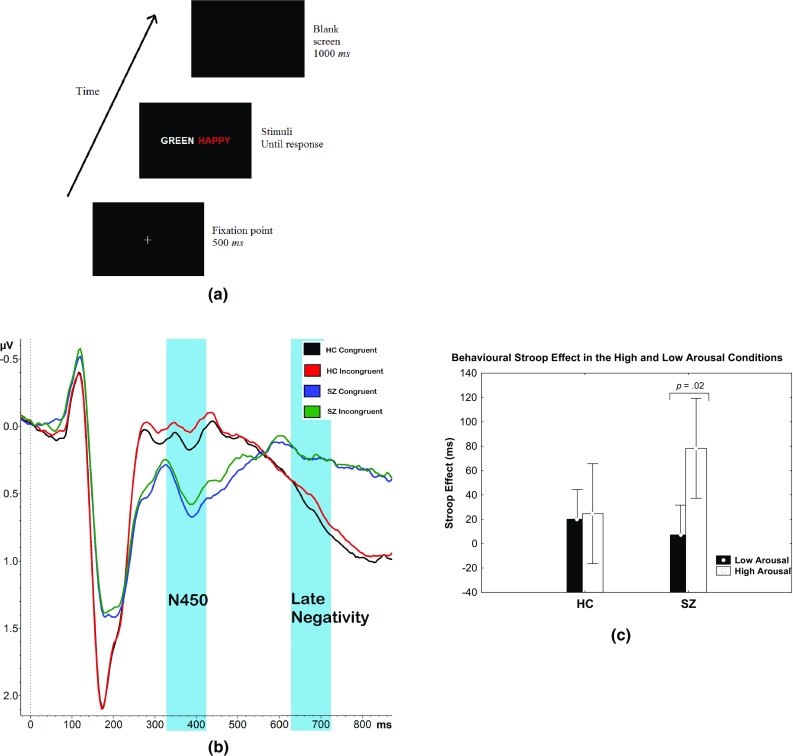
**a** A schematic illustration of the emotional Stroop paradigm, adapted from Chajut et al. (2010). **b** The ERP grand average wave at pooled fronto-central electrodes in the incongruent and congruent conditions for HC subjects and incongruent and congruent conditions for SZ subjects across all emotional conditions. Time frames highlighted in blue are the N450 (326–426 ms) and late negativity (626–726 ms) windows. **c** The behavioural arousal × congruence effect with significantly higher Stroop effect in the high arousal in contrast to the low arousal condition in SZ subjects. (**a** Reprinted, with permission from Feroz et al. (2017). Copyright 2017 with permission from Springer)

A total of 768 stimuli, delivered in six blocks (low-arousal neutral, high-arousal neutral, low-arousal positive, high-arousal positive, low-arousal negative and high-arousal negative) were presented in a pseudo-random order with the condition that no colour appeared twice in succession. Each block contained 16 different emotional words. These words were divided in two groups of eight words. Each word group was allocated two different colors (either red and blue or green and yellow). Thus, in each block there were 2 × (2 (colors) × 2 (congruence) × 2 (positions) × 8 (words)) = 128 stimuli. Participants were instructed to respond to the ink colour of the emotional word as fast and as accurate as possible by pressing one of the four horizontally aligned keys, standing for the four ink colours. The stimulus display in each trial was response terminated. The next display appeared 1500 ms after the previous response. Figure 1a illustrates the emotion–cognition Stroop paradigm used in the study.

### EEG Recording

The EEG recording was acquired using Brain Vision version 1.20 (Brain Products, Munich), conducted in a sound-attenuated and electrically shielded room. Participants were seated on a slightly reclined chair facing a 19″ computer monitor in a dark room with the eyes and the monitor distanced approximately 1 m apart. Continuous EEG activity was recorded using Ag/AgCl electrodes mounted in a 64 channel actiCAP system. Electrodes were positioned in an extended 10/20 system with the additional positions: AF7, AF3, AF4, AF8, F5, F1, F2, F6, F10, FT9, FT7, FC3, FC4, FT8, FT10, C5, C1, C2, C6, TP7, CPz, TP8, P5, P1, P2, P6, PO3, POz, PO4. Eye movements were recorded by two horizontal EOG channels positioned at the outer canthi of the left and right eye and two vertical EOG channels, one below (infraorbital) and one above (supraorbital) the right eye. During the recording, all electrodes were referenced to FCz while AFz served as the ground. Data were then collected at a rate of 1000 Hz with the impedances being kept below 5 kΩ for all recordings.

### EEG Pre-processing

EEG data were band pass filtered (0.3–30 Hz) and down-sampled to 250 Hz. Upon automatic detection (amplitude criterion of ± 80 µV) and verification by a visual inspection, intervals containing movements and muscle artifacts in any EEG channel were excluded from further analysis. Eye movements and blinks were corrected with ICA. Epochs of 1700 ms (200 ms pre to 1500 ms post-stimulus) were created for each condition after re-referencing to common average reference and were then evaluated for the ERP analysis. Finally, a baseline correction with a period of 150 ms before stimulus was performed. EEG pre-processing was performed using Brain Vision Analyzer version 2.0 (Brain products, Munich).

### Statistical Analysis

In order to comply with the sphericity requirement of the repeated measures ANOVA, the adjusted Greenhouse-Geisser correction to the univariate repeated measures ANOVA *p* values, the unadjusted degrees of freedom and epsilon values were reported throughout this paper. All multiple comparison tests were conducted using Bonferroni t given it being robust to violations of sphericity (Maxwell 1980) and its applicability regardless of significance of the *F* test (Games 1971; Wilcox 1987; Hancock and Klockars 1996; Howell 2013). The statistical analyses of this study utilised the STATISTICA 8.0, SPSS version 20 and MATLAB R2013b. Moreover, all bar graphs reported the 95% confidence interval (Altman et al. 1983).

### Behavioural Data

Mean RT and error rates were computed for each subject. The RT and accuracy data were subjected to repeated measures, mixed-design ANOVA, with stimulus type as the within-subjects factor [with factors valence (positive, neutral, negative), arousal (low, high) and congruence (congruent, incongruent)] and group (SZ, HC) as the between-subjects factor.

### Event-Related Potentials

ERPs for correct response trials (after artifact removal) were averaged for each subject and condition, namely: valence (neutral, positive and negative), arousal (low and high) and congruence (congruent and incongruent). The number of trials per condition ranged from 40 to 64 (refer to Online Appendix 1 for the number of ERP trials used for each subject and condition). They did not differ significantly (main effects or interactions) across the various conditions [group effect; *F*_1,38_ = 2.80, partial η^2^ = 0.07, *p* = .10].

The ERP effects that are of particular interest of this study were the fronto-central negative deflection in incongruent compared to congruent trials across all levels of valence and arousal. The mean pooled amplitude of the fronto-central electrodes [FC5, FC1, C3, Cz, FC2, FC6 and C4, cf. Hanslmayr et al. (2008)] was used as the dependent variable. Based on Feroz et al. (2017), two Stroop time windows that modulated valence and arousal in HC subjects were selected for congruence analysis: the N450 (326–426 ms; peak at 404 ms) and the late negativity (626–726 ms; peak at 676 ms) as illustrated in Fig. 1b.

Furthermore, the P200 (139–189 ms; peak at 164 ms) and late positive component (LPC) (791–841 ms; peak at 816 ms) time windows were investigated (refer to Online Appendix 2 for the statistical analysis results). The P200 and LPC windows were defined as ± 25 ms from the two highest peaks amplitude of the grand-average difference wave of the HC and SZ groups.

The mean amplitudes at each window (N450, late negativity, P200 and LPC) at the pooled fronto-central electrodes for each stimulus type (congruence, valence, arousal) were calculated and analysed with repeated measures, mixed-design analysis of variance (ANOVA).

### sLORETA Region of Interest (ROI) Current Density

The intention of conducting ROI source localisation analyses was to determine the brain regions involved in emotion–cognition deficiencies in SZ subjects. Source localisation analyses were conducted with LORETA KEY (version 20-02-2017), provided by the KEY Institute for Brain-Mind Research University Hospital Psychiatry, Zurich at https://www.uzh.ch/keyinst/loreta. The standardised low resolution brain electromagnetic tomography (sLORETA) method is a discrete, three-dimensional (3D) distributed, linear, and weighted minimum norm inverse solution that has the lowest possible localisation error to test point sources (Pascual-Marqui 2002). sLORETA solutions for each participant and condition were computed with a realistic head model (Fuchs et al. 2002) within the source space (6239 voxels at a resolution of 5 mm) (Jurcak et al. 2007), restricted to cortical grey matter and hippocampi, as determined by the probabilistic Talairach atlas (Lancaster et al. 2000). The current density is computed as the linear, weighted sum of the scalp electrical potentials (unit in A/m^2^).

In the event that the sLORETA current density data violated the normality assumption (assessed with Kolmogorov–Smirnov), results were transformed using natural log transformation prior to conducting any statistical analysis (Miyanishi et al. 2013). Based on the hypotheses of this study, two ROIs were selected, namely the dACC and rvACC adapted from Pizzagalli et al. (2006) and created by including all voxels with coordinates corresponding to the respective Brodmann areas (80 voxels for each ROI) (Feroz et al. 2017). As such, the scope of this study comprises of the current density analysis within the two ROIs at the time windows as discussed in “Event-Related Potentials” section.

## Results

### Behavioural Data

#### Error Rates

Significantly higher error rates were found in SZ subjects in comparison to HC subjects [*F*_1,38_ = 7.59, partial η^2^ = 0.17, *p* < .01]. There was also a significant congruence × group effect [*F*_1,38_ = 4.51, partial η^2^ = 0.11, *p* < .04], however post-hoc test did not reveal any significant difference. All analyses performed henceforth exclude error responses (Chajut et al. 2010).

#### Behavioural Analysis

We found a significant behavioural Stroop effect across the groups [*F*_1,38_ = 13.68, GG Epsilon = 1.00, partial η^2^ = 0.26, *p* < .01]. Congruent trials were responded faster (1164.54 ms, SE 88.27) compared to incongruent trials (1197.07 ms, SE 91.23) across all emotional conditions. The significant behavioural Stroop effect demonstrated conflict elicited by task.

Mixed ANOVA revealed an arousal × congruence × group effect, indicating that SZ subjects were significantly slower in the incongruent (1574.59 ms, SE 131.55) as compared to the congruent items (1496.30 ms, SE 121.52; *F*1,38 = 4.50, GG Epsilon = 1.00, partial ƞ^2^ = 0.11, *p* = .04; *T*[*df* = 38] = 12.09, *p* < .01) in the high arousing condition with a Stroop effect of 78.29 ms, as illustrated in Fig. 1c. This results in a 219% increased Stroop effect in SZ subjects compared to HC subjects. This impairment, however, was not found in HC subjects (mean RT high arousal incongruent condition: 871.40 ms, SE 131.71; mean RT high arousal congruent condition: 846.83 ms, SE 121.52).

### Sensor Level

#### Congruence Effect

**Fig. 2:**
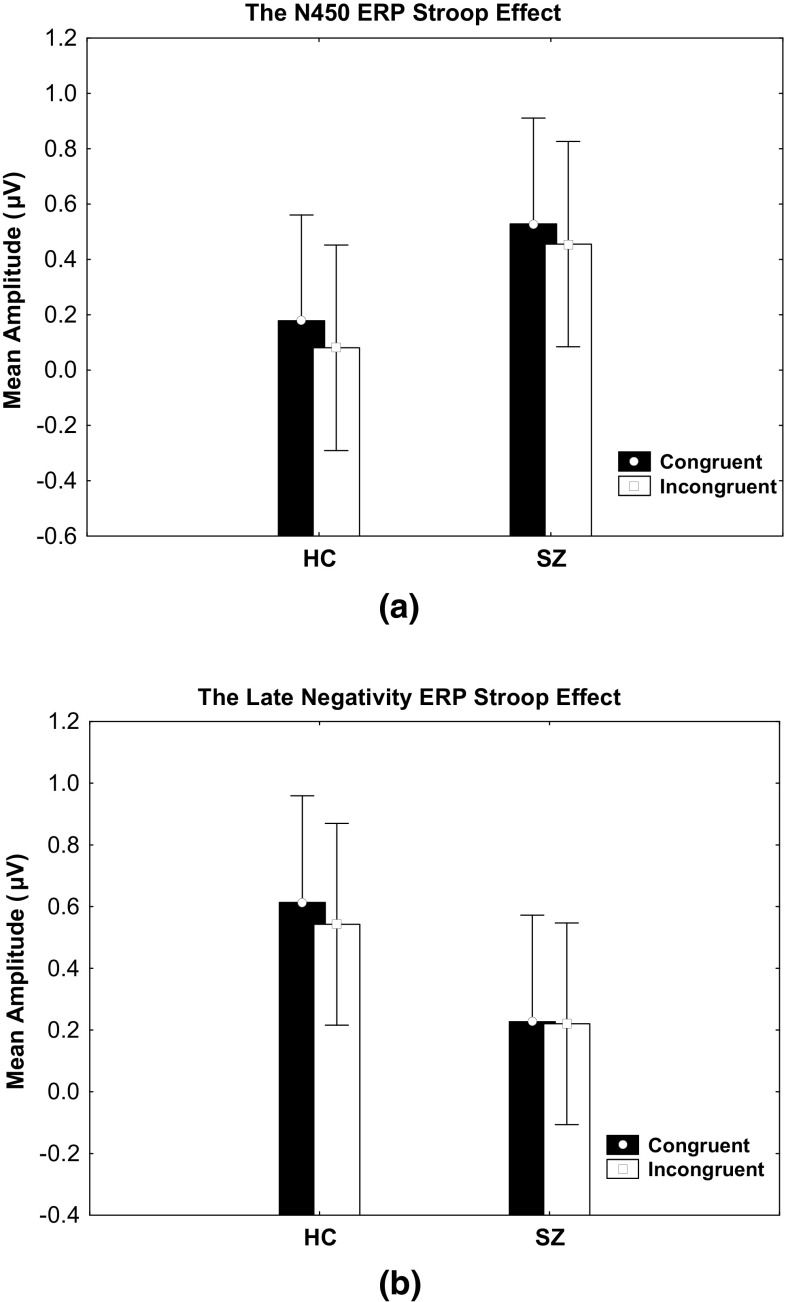
ERP responses describing the ERP Stroop-related components at the N450 and late negativity windows in HC and SZ subjects. **a** Mean ERP at the pooled fronto-central electrodes calculated from 326 to 426 ms indicating stronger negativity (decreased amplitude) in the incongruent (white) as compared to the congruent (black) items in both groups of subjects. **b** Mean ERP at the pooled fronto-central electrodes, calculated from 626 to 726 ms indicating a stronger negativity (decreased amplitude) in the incongruent (white) compared to the congruent (black) items in HC subjects, contrary to the SZ subjects. The difference, however, was statistically non-significant

The mixed ANOVA for the N450 time window revealed a significant congruence effect with increased fronto-central negativity at the N450 time window for the incongruent items in contrast to congruent items, as summarised in Online Appendix 3. The stronger N450 in the fronto-central regions for the incongruent trials (relative to the congruent trials) at this window would suggest Stroop interference, and therefore labeled with the N450 effect. This ERP effect is calculated as incongruent minus congruent difference potentials, resulting in less amplitude in the incongruent relative to the congruent items in the HC and SZ groups. As such, at the N450 window, there was an increased fronto-central negativity in HC subjects with an N450 effect of − 0.09 ± 0.03 µV, compared to SZ subjects with an N450 effect of − 0.07 ± 0.03 µV. A 22% decreased N450 effect in SZ subjects, in comparison to HC subjects was observed. Figure 2a illustrates a bar graph of the N450 effect where incongruent trials produced less mean amplitude at the fronto-central pooled electrodes compared to incongruent items. This effect was found in both subject groups.

The mixed ANOVA for the late negativity time window revealed a trend towards a statistically significant congruence effect. This ERP Stroop-related component revealed an increase in fronto-central negativity for the incongruent trials relative to the congruent trials. At the late negativity window, stronger fronto-central negativity was observed in HC subjects with a Stroop effect of − 0.07 ± 0.03 µV, compared to SZ subjects with a Stroop effect of − 0.006 ± 0.03 µV. Although there was a 91% decrease in the late negative component effect in SZ subjects compared to HC subjects, the difference was statistically non-significant. The detailed ERP statistical results are summarised in Online Appendix 3. Figure 2b illustrates the ERP Stroop congruence effects at the late negativity window, featuring less mean amplitude in the incongruent trials, compared to congruent trials in HC subjects but not SZ subjects. This effect, however, was not significant.

#### Valence × Congruence × Group Effect During N450

**Fig. 3:**
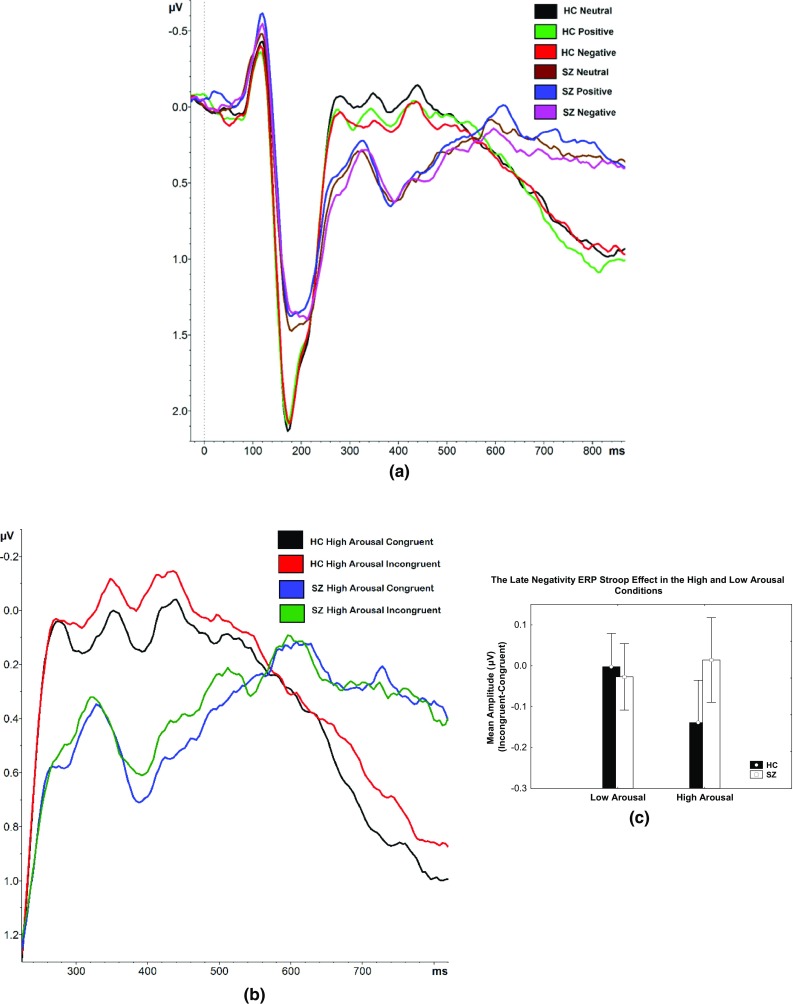
The valence ERP modulation effects. **a** The mean ERP wave at the pooled fronto-central electrodes with respect to valence (neutral, positive and negative conditions) for HC and SZ subjects. The arousal × congruence ERP modulation effects. **b** The ERP average wave at the pooled fronto-central electrodes in the high arousal congruent and incongruent condition for the HC subjects and SZ subjects. The difference between the groups is prominent within the late negativity window. **c** Bar graph of the mean ERP at the pooled fronto-central electrodes calculated from 626 to 726 ms during late negativity showed higher negative deflection in the incongruent compared to the congruent trials in the high arousal condition in the HC subjects in comparison to the SZ subjects. The difference, however failed to gain significance

As described in Online Appendix 3, the valence × congruence × group effect showed a trend towards significance at the N450 window. Post-hoc Bonferroni t revealed significantly higher mean amplitude in the positive congruent condition (t(39.34) = 2.021, *p* = .047; 0.25 ± 0.20 µV) compared to the neutral incongruent condition (0.04 ± 0.21 µV) in HC subjects. There were no significant differences found in SZ subjects. An illustration of the ERP waves for each valence condition is provided in Fig. 3a.

#### Arousal × Congruence × Group Effect During Late Negativity

At the late negativity window, a trend towards a significant arousal × congruence × group effect was observed (refer to Online Appendix 3 for the statistical details). As illustrated in Fig. 3b, c, at the late negativity window, the Stroop effect in the high arousal condition was stronger in HC subjects (− 0.14 µV, SE 0.05) compared to SZ subjects (0.01 µV, SE 0.05), although the post-hoc Bonferroni t failed to gain significance.

### ROI Time Course Analysis Using sLORETA

**Fig. 4:**
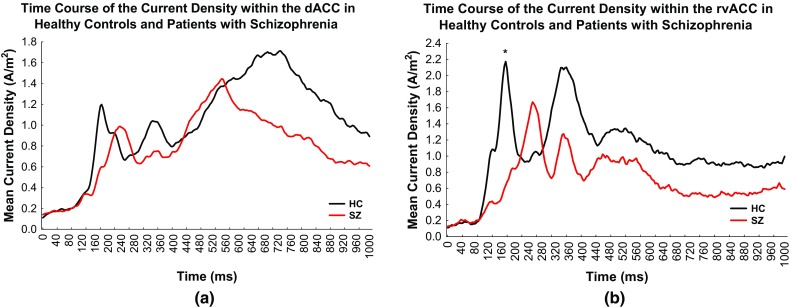
sLORETA current source density time course within the **a** dACC and **b** rvACC in HC subjects and SZ subjects. Stimulus onset was at time = 0 ms. Asterisk indicate significant less activation within the rvACC at 172 ms

Slower increase in current density was observed within both the dACC [mean difference = 47.6 ms, t(38) = − 2.22, *p* = .06 corrected] and rvACC [mean difference = 70.6 ms, t(38) = − 2.98, *p* < .01 corrected] in SZ subjects. SZ subjects also demonstrated less activation within the dACC [mean difference = − 0.60 ln A/m^2^, *F*1,38 = 4.50, partial ƞ^2^ = 0.11, *p* = .08 corrected] and rvACC [mean difference = − 0.96 ln A/m^2^, *F*1,38 = 7.02, partial ƞ^2^ = 0.16, *p* = .02 corrected] at the first peak of activity within the dACC (180 ms) and rvACC (172 ms) (refer to Fig. 4a, b for illustration).

#### Valence Modulated the N450 rvACC Activity in HC but Not in SZ Subjects

**Fig. 5:**
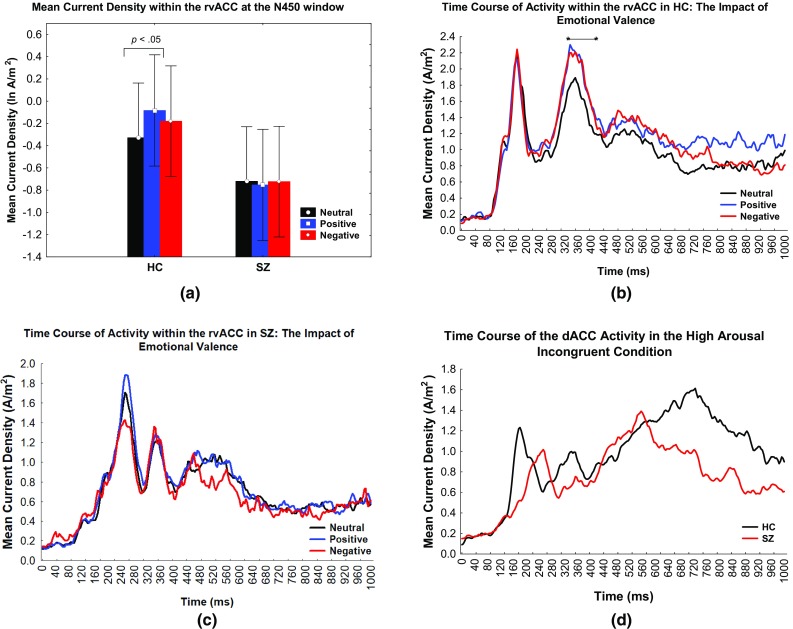
The valence current density modulation effects in HC but not SZ. **a** The mean current density within the rvACC at the N450 window in HC and SZ subjects in the neutral, positive and negative conditions. **b** Averaged time course of the current source density within the rvACC in the neutral, positive and negative conditions in HC subjects. The asterisks indicate a significant valence × group effect at the N450 window. **c** Averaged time course of the current source density within the rvACC in the neutral, positive and negative conditions in SZ subjects. The current density arousal × congruence effect in SZ. **d** Averaged time course of the current source density within the dACC in the high arousal conflict condition in HC subjects and SZ subjects

At the 326–426 ms window, a significant valence × group effect was observed [*F*2,76 = 3.61, GG epsilon = 0.98, partial ƞ^2^ = 0.09, *p* = .03]. Bonferroni t revealed that in the HC group, within the rvACC region, the mean current density was significantly higher in the positive compared to the neutral condition (t(40.35) = 2.021, *p* = .02). This effect was not significant in the SZ group (*p* = 1.00). The difference in valence modulation effect in HC subjects against SZ subjects is apparent in Fig. 5a, which illustrates the bar graph of the mean current density within the rvACC at the N450 window in all valence conditions in both the HC and SZ groups. Figure 5b, c feature the time course of the rvACC activity in HC subjects and SZ subjects, respectively. Increased rvACC activity in the positive condition compared to the neutral condition at the N450 window is visible in the HC group, contrary to that of the SZ group.

#### Decreased dACC Activity in the High Arousal Incongruent Condition During Late Negativity in SZ but Not in HC Subjects

The time course of the impact of the high arousal conflict condition on dACC activity portrayed in Fig. 5d revealed lower mean current density in SZ compared to HC subjects at the late negativity window. The high arousal conflict difference between groups showed a trend towards significance at the peak of the difference, 748 ms (*t*(38) = 1.79, *p* = .08). At this time point, patients with SZ showed lower dACC activity in the high arousal incongruent condition in comparison to HC subjects.

Group differences revealed that within the dACC region, an arousal × congruence effect existed, trending towards significance in the SZ group (*t*(39.24) = 2.023, *p* = .08) whereas the HC group resulted otherwise(*t*(39.23) = 0.32, *p* = .35). Within the late negativity window, in conflict conditions, the mean current density in the low arousal condition was higher compared to the high arousal condition in SZ subjects.

#### ROI Current Density Correlates

##### Mean dACC at Late Negativity and Mean RT Negative Correlation in the High Arousal Incongruent Condition

In the SZ group, a significant negative correlation was found between mean RT in the high arousal incongruent condition and current density within the dACC during late negativity (*r* = − 0.50, *p* = .024). See Online Appendix 4(A) for the scatterplot. This result confirms that increased current density within the dACC during late negativity is associated with decreased RT in the high arousal incongruent condition.

##### Mean dACC (Differential Responses to High and Low Arousal Conflict Condition) at Late Negativity and Accuracy Positive Correlation in HC Subjects

The differential dACC responses of the high arousal incongruent and low arousal incongruent positively correlated with behavioural accuracy in the HC subjects. The result had a trend towards significance after Bonferroni correction (*r* = 0.54, *p* = .09 corrected). As illustrated in Online Appendix 4(B), higher dACC recruitment in the high arousal condition was associated with improved behavioural performance, relative to the low arousal condition.

##### Mean dACC (Differential Responses to High and Low Arousal Conflict Condition) at Late Negativity and the Five Factor PANSS Correlation in SZ Subjects

In the SZ subjects, we observed initial significant correlation (*r* = − 0.47, *p* = .048 uncorrected) between the differential dACC responses to the high and low arousal incongruent condition and the PANSS negative factor scores. Patients with decreased dACC recruitment in the high arousal compared to the low arousal incongruent condition during late negativity had higher PANSS negative symptoms, as illustrated in Online Appendix 4(C). However, the result was statistically non-significant after Bonferroni correction (*p* = .288 corrected).

In addition, results were statistically non-significant for the positive factor (*r* = − 0.11, *p* = 4.06 corrected), disorganized factor (*r* = − 0.06, *p* = 4.96 corrected), excitement factor (*r* = − 0.12, *p* = 3.81 corrected) and emotional distress factor (*r* = − 0.39, *p* = .92 corrected).

## Discussion

Abnormal emotion–cognition interactions may be critically involved in the pathophysiology of SZ. Current research has shown that exacerbated arousal dysregulation within the dACC is associated with the cognitive impairment in patients with SZ. Nevertheless, the time window of the deficiency has yet been attained. Furthermore, the impact of emotional valence in modulating cognition and the rvACC activity in SZ subjects remains unclear. In addressing the gaps and conflicts of previous studies, this study compared the RT, ERP and sLORETA ROI current density within the dACC and rvACC at the N450 and late negativity windows of the modified emotional Stroop task of HC and SZ subjects.

### Hypoactivation of the dACC Activity During Late Negativity in the High Arousing Condition

In accordance with the hypothesis of this study, hypoactivation of dACC activity was discovered at the late negativity window (626–726 ms) in the high arousal conflict condition with concomitant cognitive deficits in SZ subjects. This hypoactivation suggests that SZ subjects may have faced deficit during the response conflict resolution. In these subjects, lower underlying current density within the dACC was found to be interrelated with higher RT in the high arousal conflict condition. Plausible differences between HC and SZ subjects were identified within the late negativity window in two instances: (1) the ERP in the high arousal incongruent relative to the high arousal congruent condition which illustrated higher ERP Stroop effect in HC relative to SZ subjects, therefore indicating that the maximization of the fronto-central regions would resolve conflict in HC subjects within this duration; and (2) a general decline of activity within the dACC starting from 560 ms and persisting to the late negativity stage for SZ subjects in the high arousal conflict condition. EEG and fMRI (Phillips et al. 1999; Williams et al. 2004) studies confirm the reduced ACC activity in the high arousal condition in contrast to the low arousal condition in SZ subjects. The results of the study bridged the gap between fMRI findings from Dichter et al. (2010), which linked dACC hypoactivation in aversive condition with cognitive impairment, as well as the findings from Kaneko et al. (1992), Williams et al. (2004) and Das et al. (2007) which linked the dysregulation of emotional arousal within the dACC, associated with cognitive deficits in SZ subjects. FMRI is inherently limited concerning its temporal resolution and therefore does not allow a precise determination of the moment of activity of the brain regions involved.

In HC subjects, the increase of processing resources within the dACC during late negativity engaged by the high arousal emotional words were found to enhance task performance. Within the same time window, we found an existing arousal–cognition interaction impairment among the SZ subjects. While being engaged with the high arousing emotional words, these patients utilised less processing resources within the dACC therefore resulting in an increased RT in the high arousal conflict condition. Findings of the current study support fMRI findings from Dichter et al. (2010) with aversive stimuli and Nelson et al. (2015) with pleasant stimuli. Interestingly the inadequate emotion–cognition integration and interaction within the ACC could potentially result in reduced goal directed behavior, leading to cognitive deficits in patients with SZ (Yücel et al. 2003). Hence, the results of the study would be consistent with the neural diathesis–stress model (Nuechterlein and Dawson 1984; Walker and Diforio 1997; Pruessner et al. 2017) which hypothesised the HPA axis dysfunction to heighten cognitive impairments in patients with SZ. The findings of this study may also be in line with the DA hypothesis of SZ associating prefrontal hypodopaminergia (Howes and Kapur 2009) with hypofrontality, found to be linked with the hypoactivation of the ACC in SZ subjects (Mientus et al. 2002) and cognitive deficits in SZ.

Remarkably, the results of this study would be in line with the idea that SZ subjects may require significantly higher RT in comparison to HC due to their struggle to inhibit irrelevant responses in the high arousing condition. The struggle may be a result of the narrowing attention (Kahneman 1973), searching for the meaning of their arousal (Clamor et al. 2015), increased lability of attention allocation (Kahneman 1973) and failure to disengage from the emotional stimuli. The battle of inhibiting responses to the task-irrelevant high arousing word may have also caused weaker ability to inhibit (Braver et al. 1999) the incongruent colour word in this category of SZ subjects. Results from this study highlight that SZ subjects with decreased dACC activity during late negativity face cognitive deficiencies during conflict in the high arousal items.

### The Impact of Emotional Valence on rvACC Activity

Emotional valence modulated both the ERP and rvACC activity within the N450 window in HC subjects but not in SZ subjects. The non-significant N450 valence impact may have been a result of increased neural activations to neutral stimuli in SZ subjects (Murray et al. 2008; Habel et al. 2010a) (also see Potvin et al. (2016) for a review). It is also important to note studies that show higher levels of arousal (Llerena et al. 2012) and stronger aversion (Cohen and Minor 2010) towards neutral stimuli in male SZ subjects. This condition may lead to the impairment of the ability to discriminate relevant from irrelevant aspects of the stimuli. The findings of the current study were associated with the DA hypothesis in patients with SZ (Kapur 2003; Howes and Nour 2016) associating increased striatal DA with the aberrant assignment of salience to a stimulus (Howes and Kapur 2009). Indubitably, the disruption of emotional valence modulation within the rvACC during N450 in SZ subjects has been added to the current literature.

### Time Course: Slower Initial Activation in SZ Subjects

This study may arguably be the first report showing significantly slower initial activation and decreased peaks of activity within the dACC and rvACC in SZ subjects in contrast to HC subjects across conditions. These findings indicate timing deficits and hypoactivation within the dACC and rvACC in SZ subjects. These findings also suggest abnormal regulation of emotion–cognition circuits in SZ, leading to impaired performance. In relevance with the results of the study, the time course of fMRI hemodynamic responses graph in Dichter et al. (2010) of the anterior cingulate gyrus (ACG) in target conditions shows slower initial activation in SZ subjects. Attaining the time course activity between these regions is important because it provides the general view of the activation and underlying temporal neural mechanisms of emotion–cognition interaction in SZ subjects which is still not fully understood.

### Limitation and Future Direction

While acknowledging the outcome of the study, several limitations of the study should be noted. Firstly, factoring both valence and arousal dimensions facilitated the detection of the arousal behavioural deficiencies suffered by SZ subjects. However, it should be noted that larger sample sizes would afford increased power to detect possible valence behavioural effect, although in comparison to valence, arousal plays a more important role in determining the degree to which emotional stimuli impact cognition (Anderson 2005). Another noteworthy limitation of the study is the cognitive abilities of the HC subjects were not evaluated. Although the HC and SZ subjects were matched with respect to their education level, a comparison of the MWT-B scores would enhance experimental control in this study.

The usage of the normative ratings of the valence and arousal word stimuli without having the patient group reviewing the word stimuli and reporting their emotional experience exists as a limitation to the current study. A separate SZ group rating was not conducted particularly due to the preserved perception towards the valence and arousal levels in emotional word shown by SZ subjects in studies such as Bonin et al. (2003), Herbener et al. (2009) and Jalenques et al. (2013). Furthermore, separate valence and arousal patient ratings were not performed in several emotional Stroop studies (Demily et al. 2010; Roux et al. 2010; Wiffen et al. 2014) and word-related studies (Klumpp et al. 2010; Strauss et al. 2011) with SZ subjects. Nonetheless, it should be noted that studies such as Cohen and Minor (2010) and Llerena et al. (2012) reported differential valence and arousal related emotional experience in SZ subjects. In this regard, future studies would benefit from an enhanced experimental control by having SZ participants rating stimuli as well as their current mood state during the experiment to ensure comparable emotional experience between both groups. This should be conducted particularly following the core experiment to avoid repetition effects in the results of the experiment. Unfortunately, the effects would then be present in the ratings. Possible alternatives to stimuli ratings include methods such as arousal predisposition trait test (Clamor et al. 2015), galvanic skin response (GSR) or electrodermal activity (EDA) hyperarousal measurement in SZ subjects (Pincus and Tucker 2002; Schell et al. 2005), tracking EMG measurements (Pincus and Tucker 2002) and pulse rate measurement (Pincus and Tucker 2002).

Notably, this experiment should be extended to include patients with arousal–cognitive (e.g. borderline personality disorder) or valence-cognitive (e.g. depression) related psychiatric disorders. Implementing the noninvasive EEG-fMRI method could facilitate exploring possible network dysconnectivity between the dACC (at the late negativity window) and rvACC (at the N450 window) with brain regions such as the amygdala and Nacc that are inaccessible via EEG.

## Conclusion

This study revealed that hypoactivation within the dACC in high arousing conditions, occurring on the 626–726 ms window in SZ subjects is associated with increased RT in SZ subjects. At the 326–426 ms window, emotional valence modulated the rvACC activity in HC subjects but not in SZ subjects. The dysregulation is suspected to be associated with the aberrant salience assignment to neutral stimuli in line with the DA hypothesis in SZ subjects.

The results of this study bridges the gap of previous studies indicating hypoactivation within the dACC and dysregulation of emotional arousal within the dACC in relation to cognitive impairment in SZ subjects. This study also provides new insights into the time window of the dACC hypoactivity (late negativity) and the valence aberrant modulation within the rvACC that occured at the N450 window in SZ subjects. These findings may be relevant for understanding the neural mechanisms underlying the disturbances of SZ subjects in selecting their responses in highly-charged emotional-cognitive situations with increased needs for a top-down conflict control.

## Electronic supplementary material

Below is the link to the electronic supplementary material.

Supplementary material 1 (DOCX 56 KB)

Supplementary material 2 (DOCX 14 KB)

Supplementary material 3 (DOCX 21 KB)

Supplementary material 4 (DOCX 38 KB)
