# Supplementary material for: The Time Course of Dorsal and Rostral-Ventral Anterior Cingulate Cortex Activity in the Emotional Stroop Experiment Reveals Valence and Arousal Aberrant Modulation in Patients with Schizophrenia
Source: Brain Topogr. 2018 Oct 4;32(1):161–77. doi: 10.1007/s10548-018-0677-0 (PMC6327077; doi:10.1007/s10548-018-0677-0)
Supplement: Supplementary file 1 — Supplementary material 1 (DOCX 56 KB) [file 10548_2018_677_MOESM1_ESM.docx]

**Appendix 1**

**Number of ERP trials in used in each subject and condition**

| **Group/**  **Condition** | **ULC** | **ULI** | **UHC** | **UHI** | **PLC** | **PLI** | **PHC** | **PHI** | **NLC** | **NLI** | **NHC** | **NHI** |
| --- | --- | --- | --- | --- | --- | --- | --- | --- | --- | --- | --- | --- |
| **HC** | 60 | 60 | 62 | 61 | 52 | 50 | 55 | 57 | 62 | 63 | 64 | 63 |
| **HC** | 62 | 61 | 61 | 63 | 61 | 61 | 62 | 61 | 63 | 62 | 63 | 62 |
| **HC** | 64 | 62 | 63 | 62 | 62 | 63 | 62 | 64 | 63 | 59 | 62 | 62 |
| **HC** | 63 | 64 | 63 | 63 | 64 | 63 | 64 | 63 | 62 | 62 | 64 | 62 |
| **HC** | 63 | 62 | 61 | 61 | 62 | 63 | 63 | 62 | 63 | 62 | 60 | 59 |
| **HC** | 60 | 61 | 62 | 64 | 60 | 61 | 63 | 64 | 64 | 63 | 64 | 64 |
| **HC** | 62 | 62 | 64 | 63 | 61 | 63 | 64 | 63 | 63 | 63 | 63 | 62 |
| **HC** | 61 | 63 | 59 | 63 | 64 | 63 | 62 | 63 | 63 | 64 | 62 | 63 |
| **HC** | 62 | 64 | 63 | 62 | 62 | 64 | 61 | 62 | 63 | 63 | 61 | 62 |
| **HC** | 59 | 62 | 60 | 62 | 63 | 63 | 59 | 62 | 61 | 61 | 62 | 63 |
| **HC** | 60 | 61 | 63 | 64 | 63 | 62 | 59 | 62 | 63 | 62 | 60 | 60 |
| **HC** | 63 | 63 | 49 | 51 | 49 | 41 | 62 | 54 | 49 | 64 | 63 | 49 |
| **HC** | 64 | 64 | 64 | 64 | 64 | 64 | 64 | 64 | 63 | 64 | 64 | 63 |
| **HC** | 64 | 63 | 63 | 63 | 64 | 63 | 63 | 64 | 64 | 64 | 63 | 64 |
| **HC** | 59 | 59 | 56 | 62 | 59 | 58 | 60 | 61 | 58 | 61 | 60 | 63 |
| **HC** | 61 | 64 | 64 | 62 | 63 | 64 | 61 | 64 | 64 | 64 | 62 | 62 |
| **HC** | 63 | 61 | 63 | 60 | 62 | 61 | 64 | 60 | 62 | 63 | 63 | 61 |
| **HC** | 64 | 64 | 61 | 60 | 63 | 64 | 63 | 64 | 63 | 64 | 64 | 64 |
| **HC** | 61 | 63 | 63 | 62 | 63 | 62 | 64 | 64 | 63 | 62 | 63 | 64 |
| **HC** | 50 | 49 | 63 | 63 | 62 | 63 | 58 | 64 | 62 | 59 | 61 | 63 |
| **SZ** | 61 | 58 | 60 | 60 | 61 | 63 | 62 | 62 | 51 | 58 | 57 | 60 |
| **SZ** | 62 | 62 | 62 | 61 | 63 | 61 | 63 | 60 | 62 | 61 | 62 | 61 |
| **SZ** | 62 | 61 | 58 | 62 | 63 | 60 | 61 | 62 | 61 | 61 | 61 | 58 |
| **SZ** | 63 | 64 | 63 | 63 | 63 | 62 | 60 | 61 | 64 | 62 | 63 | 62 |
| **SZ** | 63 | 64 | 64 | 60 | 61 | 64 | 63 | 63 | 64 | 64 | 64 | 64 |
| **SZ** | 63 | 64 | 62 | 61 | 64 | 64 | 63 | 63 | 62 | 62 | 62 | 61 |
| **SZ** | 60 | 60 | 60 | 61 | 58 | 56 | 63 | 61 | 63 | 61 | 60 | 57 |
| **SZ** | 60 | 58 | 62 | 57 | 59 | 59 | 59 | 62 | 60 | 57 | 59 | 57 |
| **SZ** | 63 | 64 | 64 | 60 | 64 | 64 | 63 | 64 | 63 | 64 | 64 | 64 |
| **SZ** | 63 | 64 | 63 | 63 | 62 | 64 | 63 | 62 | 62 | 64 | 64 | 62 |
| **SZ** | 62 | 61 | 61 | 62 | 61 | 62 | 62 | 63 | 63 | 63 | 63 | 63 |
| **SZ** | 61 | 62 | 58 | 56 | 63 | 50 | 55 | 54 | 57 | 55 | 45 | 40 |
| **SZ** | 59 | 60 | 61 | 62 | 62 | 59 | 56 | 59 | 61 | 60 | 61 | 62 |
| **SZ** | 60 | 60 | 56 | 61 | 57 | 53 | 56 | 56 | 52 | 53 | 61 | 60 |
| **SZ** | 56 | 62 | 59 | 59 | 62 | 60 | 59 | 56 | 61 | 58 | 56 | 60 |
| **SZ** | 62 | 63 | 58 | 55 | 61 | 57 | 62 | 60 | 62 | 62 | 60 | 59 |
| **SZ** | 61 | 61 | 63 | 63 | 62 | 62 | 62 | 62 | 61 | 63 | 60 | 60 |
| **SZ** | 61 | 62 | 60 | 59 | 61 | 57 | 59 | 60 | 64 | 61 | 58 | 59 |
| **SZ** | 59 | 54 | 56 | 61 | 61 | 61 | 60 | 60 | 60 | 60 | 62 | 59 |
| **SZ** | 63 | 60 | 62 | 63 | 63 | 60 | 60 | 63 | 58 | 61 | 60 | 61 |

List of abbreviations:

ULC: Neutral Low Arousal Congruent

ULI: Neutral Low Arousal Incongruent

UHC: Neutral High Arousal Congruent

UHI: Neutral High Arousal Incongruent

PLC: Positive Low Arousal Congruent

PLI: Positive Low Arousal Incongruent

PHC: Positive High Arousal Congruent

PHI: Positive High Arousal Incongruent

NLC: Negative Low Arousal Congruent

NLI: Negative Low Arousal Incongruent

NHC: Negative High Arousal Congruent

NHI: Negative High Arousal Incongruent

HC: Healthy Controls

SZ: Patients with Schizophrenia
