# Supplementary material for: The Time Course of Dorsal and Rostral-Ventral Anterior Cingulate Cortex Activity in the Emotional Stroop Experiment Reveals Valence and Arousal Aberrant Modulation in Patients with Schizophrenia
Source: Brain Topogr. 2018 Oct 4;32(1):161–77. doi: 10.1007/s10548-018-0677-0 (PMC6327077; doi:10.1007/s10548-018-0677-0)
Supplement: Supplementary file 2 — Supplementary material 2 (DOCX 14 KB) [file 10548_2018_677_MOESM2_ESM.docx]

**Appendix 2**

**Statistical Analysis: Overall group differences**

# Behavioural analysis

Generally, SZ subjects (1512.19 ms, SE 126.79) showed significantly higher RT compared to HC subjects (849.42 ms, SE 126.79; $F_{1,38}=13.66, , \mathrm{partial} ƞ^{2}=0.26, p<.01$).

# ERP group differences

There was a trend towards significant reduction in the P200 [$F_{1,38}$ = 2.98, partial $ƞ^{2}$=0.07, $p=.09$] and the LPC [$F_{1,38}$ = 7.53, partial $ƞ^{2}$=0.17, *p* < .01] mean amplitude in SZ subjects compared to HC subjects.

# sLoreta group differences

At the P200 window, SZ subjects produced significantly lower current density within both, the dACC and rvACC [$F_{1,38}=6.12, \mathrm{partial}ƞ^{2}=0.14, p=0.02$] in comparison to HC subjects. At the LPC window [$F_{1,38}=3.03, \mathrm{partial}ƞ^{2}=0.07, p=0.09$], SZ subjects had a trend towards significant lower current density compared to HC subjects.
