# Supplementary material for: The Time Course of Dorsal and Rostral-Ventral Anterior Cingulate Cortex Activity in the Emotional Stroop Experiment Reveals Valence and Arousal Aberrant Modulation in Patients with Schizophrenia
Source: Brain Topogr. 2018 Oct 4;32(1):161–77. doi: 10.1007/s10548-018-0677-0 (PMC6327077; doi:10.1007/s10548-018-0677-0)
Supplement: Supplementary file 3 — Supplementary material 3 (DOCX 21 KB) [file 10548_2018_677_MOESM3_ESM.docx]

**Appendix 3**

The N450 and late negativity ERP effects table

| Time Window | Effects | $F$ | GG Epsilon | $\mathrm{partial}ƞ^{2}$ | $p$ |
| --- | --- | --- | --- | --- | --- |
| N450 | Congruence | 16.35 | 1.00 | .30 | $<.01$ |
| Late negativity | Congruence | 3.15 | 1.00 | .08 | .08 |
|  |  |  |  |  |  |
| N450 | Valence × Congruence × Group | 2.65 | .89 | .07 | .08 |
|  |  |  |  |  |  |
| Late negativity | Arousal × Congruence × Group | 3.38 | 1.00 | .08 | .07 |
|  |  |  |  |  |  |
| Late negativity | Congruence × Group | 2.19 | 1.00 | .05 | .15 |
