# Supplementary material for: The Time Course of Dorsal and Rostral-Ventral Anterior Cingulate Cortex Activity in the Emotional Stroop Experiment Reveals Valence and Arousal Aberrant Modulation in Patients with Schizophrenia
Source: Brain Topogr. 2018 Oct 4;32(1):161–77. doi: 10.1007/s10548-018-0677-0 (PMC6327077; doi:10.1007/s10548-018-0677-0)
Supplement: Supplementary file 4 — Supplementary material 4 (DOCX 38 KB) [file 10548_2018_677_MOESM4_ESM.docx]

**Appendix 4**

**Scatterplots of significant behavioral and neural correlates**

(A) Scatterplot of the mean current density within the dACC at the late negativity window and mean RT in the high arousal conflict condition. Scatterplots of the mean differential dACC responses to high arousal incongruent relative to low arousal incongruent at the late negativity window and (B) behavioral performance (accuracy) in HC subjects (C) the PANSS negative factor scores in SZ subjects.

| ****  **(A)** | ** (B)** |
| --- | --- |
| ****  **(C)** | |
